# Unfolding Nostalgia: Spatial Visualization, Nostalgia, and Well-Being

**DOI:** 10.3390/bs15121669

**Published:** 2025-12-03

**Authors:** Maxim Likhanov, Ksenia Bartseva, Elena Soldatova, Yulia Kovas

**Affiliations:** 1 Department of Psychology, Saint Petersburg State University, 7/9 Universitetskaya Nab., 199034 Saint Petersburg, Russia; k.bartseva@spbu.ru (K.B.); e.l.soldatova@spbu.ru (E.S.); 2 Cognitive Health and Intelligence Centre, Institute for Cognitive Neuroscience, HSE University, 101000 Moscow, Russia; yvkovas@hse.ru

**Keywords:** nostalgia proneness, spatial visualization, well-being, anxiety, neuroticism

## Abstract

Research has shown that nostalgia can have psychological benefits, for example, by recreating comforting memories from the past. These memories often unfold in mental space, where one recreates events, people, objects, etc. Therefore, individual differences in nostalgic experience may relate to the ability to process spatial information. The aim of the current study was to investigate the links among spatial ability, imagery, nostalgia, and well-being. In total, 521 participants (Mage 27.7 years; SD = 12.14; 400 women) completed the following measures: Well-Being Inventory (WHO5), Neuroticism scale from BFI-2-S, Generalized Anxiety Disorder Inventory (GAD7), Southampton Nostalgia Proneness test, Nostalgia Content test, and Paper Folding—a spatial visualization test (SV). The SV did not correlate with nostalgia proneness. However, when only spatially related items were selected from the Nostalgia Content Questionnaire, the “Spatial Nostalgia Score” was positively linked with the SV and nostalgia proneness. This measure is also positively linked with well-being after controlling for anxiety (but not neuroticism). The current study provided new insights into the links between nostalgia and well-being by incorporating spatial visualization as an important element of nostalgia. Taken together, the results suggest that individual differences in the SV may be linked to spatial aspects of nostalgic experiences. This study identified directions for further measurement development and future experimental studies.

## 1. Introduction

Nostalgia is defined as an emotionally charged experience, and is linked with temporal retrospect—with memories and past events (Batcho, 2013; Wilson, 2015). However, nostalgia also unfolds in space—even the origin of the term “nostalgia” is related to homesickness (Greek nostos—homecoming, and algos—sadness) and was first used to describe the anxieties shown by Swiss mercenaries fighting far from home (Fuentenebro De Diego & Valiente Ots, 2014). Recent research also suggests a paradoxical link between nostalgia and well-being. On the one hand, it can have positive effects on well-being via improving emotional regulation, empowering feelings of connection, and protecting against feeling of loneliness by recreating some comforting memories from the past (Sedikides & Wildschut, 2019; Van Tilburg et al., 2019); on the other hand, nostalgia is weakly and positively correlated with neuroticism and anxiety (Dai et al., 2024; Seehusen et al., 2013; Tullett et al., 2015). The current study investigates the role of spatial cognition in nostalgic experiences, as well as its contribution to the nostalgia–well-being association.

### 1.1. Nostalgia and Space

Nostalgia is often spatial in nature, with memories of the past directly linked to particular spatial locations and involving spatial visualization. It is possible that different people are more prone to spatial vs. other types of “nostalgizing” (e.g., verbal). Indeed, previous research has suggested that triggers and contents of nostalgia might be either qualitatively different (e.g., different sensory triggers “activating” nostalgia) or quantitatively different (e.g., same triggers being more or less important for some people but not for others) for different clusters of people (Hepper et al., 2014). Research assessing the content of nostalgic memories (Batcho, 1995; Hepper et al., 2014; Wildschut et al., 2006) showed that home and other important places were frequently the content of nostalgia, alongside past friends, toys, and loved ones. Such reliance on places during nostalgic memories suggests that nostalgic experiences should involve at least some spatial processing, along with memory and emotional processing.

Neuroscientific research (see Yang et al., 2022 or the review by Yang et al., 2023) shows that nostalgia activates multiple brain regions, including areas associated with self-reflection (medial prefrontal cortex, posterior cingulate cortex, and precuneus), emotion regulation (anterior cingulate cortex and medial prefrontal cortex) and reward processing (striatum, substantia nigra, ventral tegmental area, and ventromedial prefrontal cortex). In addition, these studies showed that nostalgia is linked with the hippocampus, medial prefrontal cortex, posterior cingulate cortex, and precuneus—areas associated with autobiographical/episodic memory (Gilboa, 2004; Maguire et al., 2000). In turn, this type of memory is related to personal experience for events in a spatio-temporal context (Tulving, 1983).

In particular, the involvement of the hippocampus in nostalgia may explain why place and time are intertwined in nostalgia. Research converges on the crucial role of the hippocampus in spatial and topographic memory (Abrahams et al., 1999; Maguire et al., 1996, 1998; Maguire & Mummery, 1999; O’Keefe & Nadel, 1978; Spiers et al., 2001; Vargha-Khadem et al., 1997). Both behavioral and neurological data agree on a shared basis of spatial and episodic memories, with spatial context acting as a scaffolding structure for individual past events (Robin, 2018). In addition, the hippocampus plays a role in encoding emotional memories (Robin et al., 2018) and encoding temporal information (coming to it from the frontal lobes), serving as a timestamp for when the individual visited this location. For example, one recent study (Tsao et al., 2018) has linked the entorhinal cortex—a key area in spatial orientation and navigation, along with the hippocampus (Fyhn et al., 2004; Hafting et al., 2005; Sargolini et al., 2006)—to time perception. Tsao and colleagues suggested that the representation of time, divided into episodes, is integrated with spatial information recorded in the middle entorhinal cortex, which allows the hippocampus to store a single representation of what, where, and when. Consistent with this, research has shown that impairments in both spatial and episodic memory are often the first symptoms of Alzheimer’s disease (Kolb & Whishaw, 1996).

Further, Rubin and colleagues have outlined a multiple-systems model of autobiographical memory, in which autobiographical memories consist of a number of components that are behaviorally and neuropsychologically distinct, including sensory imagery, emotion, and narrative coherence (Rubin, 2006)—similar to nostalgic experience that also includes such components (Yang et al., 2023). In particular, research suggests that visual imagery plays a fundamental role in autobiographical memory (Greenberg & Knowlton, 2014), distinguishing two types of imagery: visual–object imagery—visualizing pictorial appearances of objects and scenes in terms of their shape, color, brightness, and texture; and visual–spatial imagery—visualizing spatial relations and movements of objects and their parts, and spatial transformations (Blajenkova et al., 2006). These two types of imagery correlate to a degree and have a partially overlapping neural network composed of occipitotemporal (ventral “visual–object” pathway) and occipitoparietal (dorsal “visual–spatial” pathway) regions, and also by the dorsolateral prefrontal cortex/frontoparietal control network (related to working memory and executive functions) (Blazhenkova, 2016; Blazhenkova et al., 2025; Kozhevnikov et al., 2005; Mazard et al., 2004).

### 1.2. Nostalgia and Individual Differences in Spatial Ability

Since space is so fundamental to nostalgia, it is possible that individual differences in spatial ability are linked to individual differences in nostalgic experiences. Spatial ability (or spatial abilities; see some discussion on the spatial ability structure (M. Likhanov et al., 2022; Lohman, 1979; Uttal et al., 2013)) is the ability to process information about place and space (Lohman, 1996). In particular, people with greater spatial abilities might have better visual imagery and more detailed episodic/autobiographical memory (Burgess et al., 2002; Moscovitch et al., 2006; Stella et al., 2012), and therefore have more proneness to experience nostalgia and/or more vivid nostalgic experiences. Indeed, previous research showed that details of a memory unravel in mental space (Maguire et al., 2016), and that richer spatial context at encoding may lead to more vivid, long-lasting recall (Chang et al., 2024). It is also possible that people with higher spatial ability will benefit more from nostalgic experiences (in terms of well-being gains or reductions in anxiety; Frankenbach et al., 2021; Leunissen et al., 2021) due to them having a greater capacity to recollect specific details.

In this context, a parallel can also be drawn with the mechanisms proposed to explain the link between individual differences in mathematical and spatial abilities. One of the potential causes of this link is that spatial visualization could act as the “mental blackboard” on which calculations take place (“mental simulation”) (Hawes & Ansari, 2020; M. Likhanov et al., 2024b). It is possible that spatial abilities act as the same mental space “in which” nostalgic experiences unfold.

### 1.3. Nostalgia and Well-Being

Research has shown that nostalgia is positively correlated with subjective well-being (Luo et al., 2019), and this relationship with well-being is strengthened once neuroticism is controlled for. The same study also demonstrated that individuals carrying the short allele of the 5-HTTLPR gene—which is associated with heightened sensitivity to negative experiences—exhibited greater nostalgia proneness, with neuroticism mediating this relationship. Therefore, the positive effects of nostalgia on well-being could be masked by a greater frequency of nostalgizing in people with higher neuroticism. Consistent with this, a recent meta-analysis found that individuals with both high and low levels of neuroticism are similarly likely to experience psychological benefits from engaging in nostalgic reverie (Frankenbach et al., 2021).

In contrast, some research indicated that individuals with a habitual tendency to worry may experience heightened anxiety and depression when engaging in nostalgic reminiscence, potentially leading to rumination and distress (Verplanken, 2012). In addition, research has shown that nostalgia can have negative consequences for well-being in displaced populations, probably because it undermines optimism for the future (Agha, 2019; Wildschut et al., 2019). It remains unclear whether neuroticism can indeed interfere with certain psychological benefits derived from nostalgia.

### 1.4. The Current Study

The current study is the first to investigate the link between spatial ability (spatial visualization) and nostalgic experiences from an individual differences perspective. The three main aims are as follows: (1) to identify nostalgia types according to predominant nostalgia triggers and content (spatial vs. other); (2) to investigate whether higher spatial ability is associated with greater nostalgia and well-being; and (3) to investigate the nostalgia–well-being link: whether neuroticism and anxiety may modulate the positive effects of nostalgia on well-being.

In order to investigate this, we used a new instrument (developed by the study team; Alenina et al., 2023) that aimed to tap into the content of nostalgic experiences (visual images, tastes, scents, etc.), triggers of nostalgia (specific places, tastes, sounds, images, or sadness), and the presence of other people (friends, family, etc.)—Nostalgia Content Questionnaire. 

We then conducted Latent Class Analysis (Slominski et al., 2024), which allows for inferring the profiles from categorical/nominative data (see the growing body of research in the area of motivation, e.g., Fiévé et al., 2025) to investigate whether there are any groups of participants that share similar nostalgic experiences; for example, whether a spatial visualization class could be inferred that is different from other specific sensory classes (auditory- or odor-based; Reid et al., 2015), or classes in which nostalgia is triggered by meeting with friends or just feeling sad (Sedikides & Wildschut, 2022; Wildschut et al., 2006).

After that, we investigated whether these profiles could be used to predict spatial ability and nostalgia proneness. We used the Paper Folding test—a test tapping into the visualization component of spatial ability (M. Likhanov et al., 2022; Rimfeld et al., 2017)—which should presumably serve as a mental blackboard for nostalgizing, similar to math cognition (M. Likhanov et al., 2024b). Our expectation was that participants who endorse a particular class could demonstrate higher or lower spatial ability, nostalgia proneness, and well-being.

In addition, we selected all items that concerned spaces, places, or images from the Nostalgia Content Questionnaire and created a new “Spatial Nostalgia Scale” by summing up these items. We then performed correlational analysis, with and without controlling for neuroticism and anxiety (see Luo et al., 2019, for discussion of the role of neuroticism in nostalgic experiences), to see whether people who are “spatially oriented” are more prone to nostalgia, have higher spatial ability and well-being, and lower anxiety.

## 2. Materials and Methods

### 2.1. Participants

In total, 521 participants (mean age 27.7 years; standard deviation 12.14 years; 400 women) took part in this study. Participants (mostly students) were recruited via Saint Petersburg State University’s research participation scheme and social networks advertising.

### 2.2. Procedure

Participants used their individual laptops and smartphones to complete 6 psychological measures as part of a larger online data collection.

This study was approved by the Ethics Committee of Saint Petersburg State University (date: 6 July 2023, number of approval: 24). All participants provided informed consent in accordance with the approved ethical protocol. Consent was collected through the study platform’s integrated consent form prior to beginning the survey.

### 2.3. Measures

#### 2.3.1. Nostalgia Content Questionnaire

This measure was developed by the authors of the current study based on the Vividness of visual experiences scale (Marks, 1973), and previous research into the triggers and content of nostalgia (Sedikides & Wildschut, 2022; Wildschut et al., 2006). The 13 questions covered triggers of nostalgia (e.g., specific places, tastes, sounds, images, or sadness); content (e.g., people, objects, places, etc.); and vividness of experience (colors, sensory modalities, etc.)). Most of the items of the questionnaire were binary, i.e., participants were to answer Yes or No. Example item: “I usually experience nostalgic memories when I smell something from the past”. Full list of items is available in Table 1. The validation information is available in (Alenina et al., 2023).

In addition to raw data, we analyzed a Spatial Nostalgia Score. To create this score, we recoded 9 items that are relevant to spatial cognition (i.e., including information about places, visual imagery, or moving in space) from the Nostalgia Content Questionnaire into dummy variables and summed them up. These items are marked in Table 1 and are in bold. The selection of these questions was carried out by a multidisciplinary expert group with backgrounds in linguistics and psychology, ensuring that the items were theoretically grounded and aligned with this study’s objectives. The resulting score varied from 0 to 12, with larger scores reflecting greater preference for space-related triggers and experiences.

In addition, the following 5 established measures were used: Nostalgia Proneness Questionnaire, Big Five Inventory (BFI), Generalized Anxiety Disorder Inventory (GAD7), Short Well-Being Inventory (WHO5), and the Spatial Visualization Ability Test—Paper Folding.

#### 2.3.2. Proneness to Nostalgia

The 7-item questionnaire (Routledge et al., 2008) is designed to measure participants’ proneness to nostalgic experiences of the past, and the value of these nostalgic experiences. The participants are instructed to rate the items on a scale from 1 (“never”) to 7 (“very often”), with a higher total score indicating higher proneness to nostalgia (i.e., more value a person assigns to nostalgic experiences and higher frequency of such experiences). Example item: “How often do you experience feelings of nostalgia?” The questionnaire was adapted to Russian (Authors, in preparation) and demonstrated good validity (Cronbach’s alpha = 0.90).

#### 2.3.3. Neuroticism Scale from the Big Five Inventory

The Big Five Inventory (BFI; John et al., 2008) assesses the following personality traits: extraversion, neuroticism, openness to experience, helpfulness, and conscientiousness. A Russian adaptation of the short second version of BFI (BFI-2-S) was used (Mishkevich et al., 2022). The questionnaire consists of 30 questions. Participants are asked to select the most appropriate statement by marking their level of agreement on a scale from 1 (strongly disagree) to 5 (strongly agree). Only questions related to the neuroticism scale were used in the current study. Example item: “I can be tense” (neuroticism). The scale demonstrated high internal consistency in previous research in Russian samples, as evidenced by high test–retest reliability and Cronbach’s alpha of above 0.65 (Mishkevich et al., 2022).

#### 2.3.4. Generalized Anxiety Disorder (GAD-7)

GAD-7 (Spitzer et al., 2006) is a seven-item self-report instrument designed to assess the presence of GAD symptoms. Participants are instructed to evaluate the extent to which the following items have reflected their experiences over the past two weeks, responding to the prompt: “How often were you bothered by the following problems in the last two weeks?” Example items include “Feeling nervous, anxious, or on edge” and “Trouble relaxing”. Responses are scored on a scale from 0 (“not at all”) to 3 (“almost every day”). A total score was computed from the sum of all the items. Higher scores indicated greater presence of GAD symptomatology. We used a Russian adaptation of this instrument, which showed high internal consistency (0.85) in a large sample of adolescents (M. Likhanov et al., 2024a).

#### 2.3.5. Well-Being—WHO5

The 5-item World Health Organization Well-Being Index (WHO-5) is a short scale measuring subjective well-being (Bech et al., 1996). The WHO-5 items are as follows: (1) “I have felt cheerful and in good spirits”, (2) “I have felt calm and relaxed”, (3) “I have felt active and vigorous”, (4) “I woke up feeling fresh and rested” and (5) “My daily life has been filled with things that interest me”. Participants rated how well each of the 5 statements applies to them when considering the past two weeks. Each of the 5 items is scored from 5 (all of the time) to 0 (none of the time). The raw score, therefore, theoretically ranges from 0 (absence of well-being) to 25 (maximal well-being). The questionnaire demonstrated high validity in the current sample, as evidenced by Cronbach’s alpha of 0.86.

#### 2.3.6. Spatial Visualization Ability Test—Paper Folding

This test aimed to assess spatial ability (Ekstrom et al., 1976). In each trial, participants saw a screen where a piece of 2D square paper was folded, and a hole was punched at the locations indicated by an arrow. Participants needed to select one of the 4 options, which corresponded to the unfolded paper sheet. The task featured 15 items with a 20 sec time limit for each item. The task was adapted from (Rimfeld et al., 2017), and showed high validity in previous studies in Russian samples, with split-half reliability equal to 0.85 (M. V. Likhanov et al., 2018).

## 3. Results

### 3.1. Data Preprocessing

Outliers were deleted using the interquartile range (IQR), i.e., [25th percentile]—1.5 × IQR and [75th percentile] + 1.5 × IQR (McGill et al., 1978). The number of outliers was very small (less than 1%), with two outliers deleted from GAD-7 and one from the Nostalgia Proneness test. Due to missing data, the number of participants varied for different traits, with Ns ranging from 440 (GAD7) to 474 (WHO5).

### 3.2. Descriptive Statistics

Descriptive statistics for the Nostalgia Proneness, Neuroticism, WHO5, Paper Folding, and GAD7 tests are presented in Table 2.

### 3.3. Nostalgia Content Profiles

Frequencies for each item of the Nostalgia Content Questionnaire are presented in Table S1 in Supplementary Materials. Some items demonstrated little variability; for example, strangers were almost never present in nostalgic experiences (6.7%), and nostalgic experiences were mostly colored (91.7%). Other items showed almost equal distributions for different options; for example, 52.4% participants perceived sounds as triggers of nostalgia, whereas 47.6% did not select this option.

#### 3.3.1. Latent Class Analysis

We employed Latent Class Analysis (LCA) to identify subgroups of respondents based on binary and categorical survey items. LCA is a clustering technique that groups individuals with similar response patterns, thereby modeling unobserved heterogeneity in the population (Slominski et al., 2024). This approach is especially appropriate for binary or categorical data, as LCA treats the observed survey responses as categorical indicators of latent classes (in contrast to latent profile analysis, which is used for continuous indicators (Sinha et al., 2021)). We fitted a series of LCA models using maximum-likelihood estimation and evaluated solutions with varying numbers of classes (from 2 to 6). The optimal number of latent classes was determined by comparing goodness-of-fit indices—including the Akaike Information Criterion (AIC), Bayesian Information Criterion (BIC), and sample-size-adjusted BIC, as well as likelihood ratio tests (e.g., Vuong–Lo–Mendell–Rubin and bootstrap LRT). In line with recent recommendations (Slominski et al., 2024), when fit indices provided conflicting conclusions, we prioritized the model that offered interpretable and theoretically meaningful classes.

For Latent Class Analysis, we excluded variable Nost_4 (“The surrounding environment in my nostalgic memories is usually: black and white”) and Nost_8 (“In my nostalgic memories, I am usually with: someone else”) because they partly overlapped in terms of content with adjacent variables (and were conditional on them)—variable Nost_4_2 (“The colors in my nostalgic memories are: warm”) and Nost_8_2 (“The people usually present in my nostalgic memories are: family or friends”), respectively, which violated the analysis assumptions. We also excluded the first question from the Nostalgia Content Questionnaire as it overlapped substantially with the frequency of nostalgic experiences and correlated quite highly with the Nostalgia Proneness Questionnaire.

**Figure 1:**
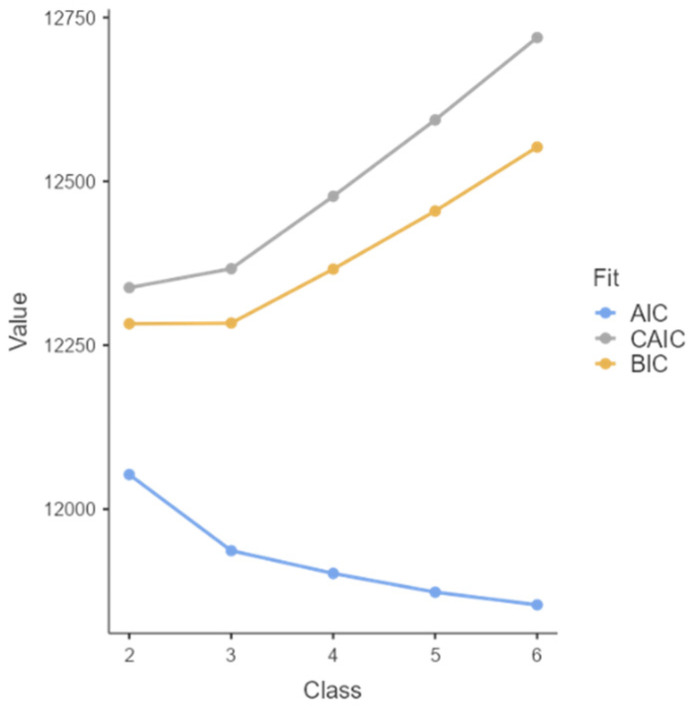
Elbow plot for model selection. Note: model with 3 classes demonstrated the following fit indices: log-likelihood = −5885; AIC = 11,937, CAIC =12,367, BIC = 12,284; entropy 0.769 and df = 400.

The elbow plot showed that the optimal number of classes is three, with the increasing number of classes leading to the fit indices becoming better indefinitely (i.e., demonstrating overfit), but not adding to the interpretability of profiles. Thus, we decided to use three classes. See Figure 1.

**Figure 2:**
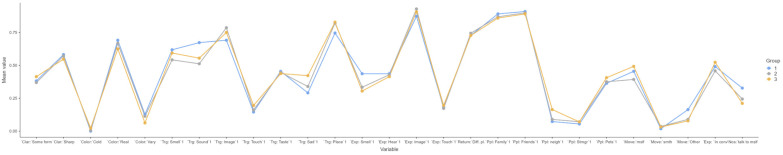
Latent profile analysis results. Note: dots on the graph illustrate the mean value of each item for a particular class; Classes are marked as group 1, 2 or 3 and are color-coded. Abbreviations: Clar—Clarity; Trg—Triggers; Exp—Experience; Diff—different; pl.—place; Ppl—people; neigh—neighbors; Strngr—strangers; mslf—myself; smth—something; in conv—in conversation; Nos—nostalgia.

The resulting profile plot (see Figure 2) demonstrates no clear separation among the three classes in terms of nostalgic experiences. There were a few small differences in mean scores among the three profiles for some items: “I usually experience nostalgic memories when: I hear sounds that remind me of the past” (Trg: Sound on the Figure 2); “I taste something that triggers memories” (Trg: Taste); “Most often during nostalgic memories, I smell something (e.g., baked goods; Exp: Smell)”; “In my nostalgic memories: I move around dynamically (e.g., inside a house; Move: mslf)” and “The space or objects move around me; Move: Other”. No “spatial” or other intuitively meaningful profile emerged.

#### 3.3.2. Profile Effects on Anxiety, Well-Being, Nostalgia Proneness, and Spatial Ability

In order to test the differences between the three profiles in anxiety, WHO-5 scores, nostalgia proneness, or spatial ability, we ran four one-way between-subject ANOVAs with profiles as IVs and the aforementioned variables as DVs (see Table S2 in Supplementary Materials). The results showed no group differences by profile (*p* < 0.05).

### 3.4. Spatial Nostalgia Score

To further investigate links between nostalgia content and spatial ability, we used Spatial Nostalgia Score (SNS), which was derived from “spatially related” items of the Nostalgia Content Questionnaire.

**Figure 3:**
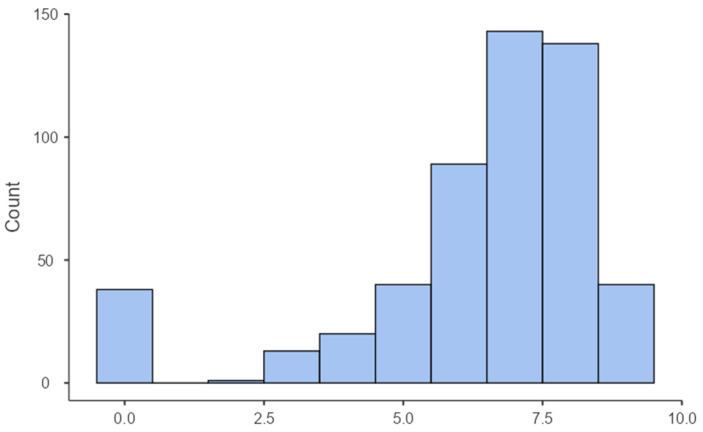
Distribution of SNS. Note: the x-axis shows the number of spatially related items selected in the SNS, and the y-axis shows how many people selected each corresponding number of items.

**Figure 4:**
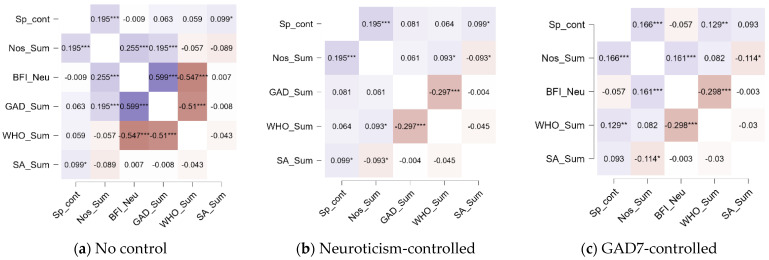
Heatmaps of correlations among the study variables, with and without controlling for GAD7 and neuroticism. Warmer, reddish colors indicate stronger positive correlations, whereas cooler, blue tones represent stronger negative correlations. Note: * *p* < 0.5; ** *p* < 0.1; *** *p* < 0.001; Sp_cont—Spatial Nostalgia Experiences Scale; Nos_Sum—nostalgia proneness; BFI-Neu—Neuroticism scale from BFI; GAD_Sum—Generalized Anxiety Disorder; WHO_Sum—WHO5 Questionnaire; SA_Sum—Spatial Ability measure—Paper Folding.

The score was bimodal (see Figure 3), with most people selecting around seven items (the mode was 7), and a small group of participants characterized by zero spatial triggers or content. We used Partial Spearman’s rho, as SNS score violated the normality assumption. The correlation analysis showed a small but positive link between spatial visualization score and this spatial content scale (Spearman’s rho = 0.099, *p* < 0.05; see Figure 4).

### 3.5. Associations Among Nostalgia and Other Study Variables 

After that we explored the links between nostalgia proneness and well-being measures. Nostalgia proneness did not show correlations with WHO5; however, it showed a modest positive correlation with neuroticism (Spearman’s rho = 0.25, *p* < 0.001) and GAD7 (0.19, *p* < 0.001), with greater neuroticism and anxiety linked with more nostalgia. After controlling for Neuroticism, following (Luo et al., 2019), a modest positive correlation (0.09, *p* < 0.05) between nostalgia proneness and WHO5 emerged—with greater well-being associated with more nostalgia. After partialling out GAD7, nostalgia proneness showed weaker correlations with Neuroticism but no correlation with WHO5.

In addition, we explored the associations between the novel score—SNS—and other study variables, with and without controlling for neuroticism and GAD7. Significant modest positive correlations emerged with nostalgia proneness (Spearman’s rho = 0.19, *p* < 0.001) and spatial ability (rho = 0.09, *p* < 0.05). No correlations emerged with WHO5, Neuroticism, and GAD7. These results did not change when we partialled out neuroticism. However, when we partialled out GAD7, we found a positive correlation between the Spatial Nostalgia Scale and WHO5 (rho = 0.12, *p* < 0.01).

Contrary to our hypothesis, spatial ability did not correlate with nostalgia proneness, although a weak negative correlation emerged with it when controlling for Neuroticism (−0.09, *p* < 0.05) and GAD7 (−0.12, *p* < 0.05). All correlations, with and without controlling for neuroticism/anxiety, are presented in Figure 4.

## 4. Discussion

The current study provided new insights into the associations between nostalgia and well-being by incorporating spatial visualization as an important element of nostalgia, and testing whether spatial ability can modulate nostalgia–well-being links. This study identified directions for further measurement development and future experimental studies.

### 4.1. Profiles of Nostalgia Experiences

The first aim of our study was to identify nostalgia types according to predominant nostalgia triggers and content (spatial vs. other). The analysis of the nostalgia content questionnaire data demonstrated no meaningful profiles. Although three profiles emerged, they were not clearly separated in terms of modality, location, presence of others, etc. There were also no differences in anxiety, well-being, nostalgia proneness, and spatial ability as a function of emerged profiles.

One possible explanation for no clear profiles emerging from the Nostalgia Content Questionnaire in Latent Class Analysis is that each person’s recollections are highly idiosyncratic: there are “marked interindividual differences in the quantity and quality” of personal memories (Palombo et al., 2018). Moreover, people tend to have stable but unique retrieval styles; for example, one study showed that features of memory recall (such as visual imagery, emotional intensity, coherence, etc.) are extremely consistent within individuals across time (Rubin, 2021). However, different sensory or contextual cues may dominate for different people. Some studies, for instance, find that odor cues produce especially rich, vivid childhood memories (the classic “Proust effect” (De Bruijn & Bender, 2018), whereas other work shows that the familiarity of a spatial context determines whether a memory is recalled in general vs. highly detailed form (Robin et al., 2019). This suggests that one person might be mostly driven by smells when remembering, another by spatial or visual cues, and so on. Because these cue–memory relationships vary continuously across individuals (rather than splitting neatly into groups), applying a latent-class or clustering analysis to the questionnaire will simply recover a diffuse, overlapping set of cases.

Another possible explanation is that the questionnaire itself may not cleanly separate distinct experience-trigger profiles. Such results were also observed for the standardized self-report instruments of autobiographical memory—probably due to such methods often having overlapping scales. For instance, the Survey of Autobiographical Memory (SAM)—a widely used questionnaire in memory research—was found to have “mixed” subscale independence: its episodic and semantic memory items did not load cleanly onto separate factors and “are not independent” measures of those constructs (Setton et al., 2022). Likewise, self-report ratings tend to capture only a fraction of the variability in actual recollection: one study reported that metacognitive memory ratings explained only 34% of the variance in recalled memory vividness (Aytürk et al., 2024). Such measurement limitations mean that clustering (Latent Class Analysis in this case) has trouble finding robust groupings—noise and construct overlap wash out any clear profiles of cue–trigger or content patterns.

### 4.2. Nostalgia and Spatial Ability

The second aim was to investigate whether higher spatial ability is associated with greater nostalgia. As nostalgia is inherently spatial, we assumed that individual differences in ability related to spatial process will underlie individual differences in nostalgia proneness. Contrary to our expectations, our spatial ability measure (Paper Folding test) did not correlate with nostalgia proneness. However, our data showed that when only spatially related items were selected from the Nostalgia Content Questionnaire, the resulting SNS was weakly positively linked with spatial ability and nostalgia proneness. Taken together, these results suggest that individual differences in spatial ability may be specifically linked to spatial aspects of nostalgic experiences.

The results are consistent with the view that nostalgic recollections draw on the same episodic-memory and scene-construction systems that underlie spatial memory. And indeed, neuroimaging studies show that retrieving personal nostalgic events engages hippocampal and default-mode regions (e.g., precuneus) involved in reconstructing past scenes (Oba et al., 2016). People who more spontaneously retrieve spatial information may have faster, richer recollection (Hebscher et al., 2018). Similarly, adults with stronger spatial memory or mental imagery may be able to “place” themselves more fully in a remembered scene, yielding more vivid, coherent nostalgic episodes. Indeed, those lacking visual imagery report fewer episodic details and weaker hippocampal engagement during recall (Monzel et al., 2024). Overall, our data showed that spatial ability could be involved in nostalgic experiences, probably as a mental blackboard where nostalgic experiences unravel (analogous to one of the accounts, explaining math–spatial ability associations; Hawes & Ansari, 2020).

### 4.3. Nostalgia and Well-Being: The Role of Neuroticism and Anxiety 

The third aim was to investigate a nostalgia–well-being association. Specifically, we expected that nostalgia is negatively associated with well-being, but this negative association may mask the positive effect of nostalgia on well-being. This is because people turn to nostalgia to buffer anxiety. In other words, neuroticism and anxiety may modulate the positive effects of nostalgia on well-being. Our data showed that nostalgia proneness did not correlate with WHO5, but correlated positively with neuroticism and anxiety. As expected after partialling out Neuroticism, it correlated positively with well-being. In addition, when we partialled out anxiety, a positive correlation between the Spatial Nostalgia Experiences Scale and well-being emerged. This is consistent with the suggestion that more detailed nostalgic memories could lead people to turn to nostalgia more often and could amplify nostalgia’s emotional payoffs (i.e., higher well-being in those who demonstrated higher spatial content scores).

Interestingly, it was GAD-7—but not neuroticism (shown in previous studies, Luo et al., 2019, but see the meta-analysis by Frankenbach et al., 2021)—that modulated the link between nostalgia and well-being in the current study. One explanation could be that the hypervigilance and intolerance of uncertainty that are at the core of anxiety (Carleton, 2016) may interfere with the reflective and meaning-making processes inherent in nostalgia. Moreover, preoccupation with threat and difficulty disengaging from worry-based cognition in highly anxious individuals (Eysenck et al., 2007), which may preclude nostalgia-triggered self-continuity, social connectedness, and meaning in life (Routledge et al., 2008, 2011). It should be noted that, in our study, the measures of anxiety and neuroticism showed a substantial correlation (0.6), with previous research demonstrating the two sharing many behavioral outcomes, such as general reactivity to stress, a tendency to feel tension, and inability to relax (Gomez & Francis, 2003; Hale et al., 2010; Papageorgiou et al., 2025). However, neuroticism is a broad personality trait, reflecting stable dispositional tendencies that span many emotional domains, whereas the GAD-7 is a symptom scale assessing the severity of clinical anxiety over the past two weeks (see discussion in Alenina et al., 2025). These differences could explain why neuroticism and anxiety showed differential patterns in relation to nostalgia, calling for further research into the role of individual differences in nostalgia–well-being links.

## 5. Conclusions and Future Directions

The current study provided some evidence on the role of spatial ability in nostalgic experiences and its connections to well-being. However, to gain further insights into these relationships, experimental studies are needed. For example, such studies can test whether the process of nostalgia is impacted by concurrent spatial tasks (e.g., by the storage of conflicting or irrelevant spatial information), which is administrated by researchers before nostalgia induction (Viviani et al., 2023). We think that such a concurrent task could either decrease the number of spatial details in a nostalgic memory or preclude nostalgizing completely due to lack of spatial resources. In addition, studies could test whether people with higher spatial ability or imagery may benefit from nostalgia interventions more than people with lower abilities. This could be tested by incorporating spatial ability measures into nostalgic experiments.

Further, future research could investigate whether “spatialized” nostalgia inductions—e.g., prompting participants to recollect more spatial details, such as places, locations of objects—would lead to more psychological benefits. These spatial elements could be added to existing nostalgia manipulations, such as inducing nostalgia with a vivid recall of past experiences vs. presenting sensory stimuli to elucidate nostalgic feelings (Wildschut & Sedikides, 2024).

Moreover, more research is needed to investigate whether nostalgia is differentially related to different facets of spatial ability. In the current study, we selected the Paper Folding test as a proxy for spatial ability, as this measure shows good psychometric properties and taps into the visualization facet of spatial ability (M. Likhanov et al., 2022; M. Likhanov et al., 2024b). However, previous research also showed that the general spatial ability factor explains around 40% of the variance, with each spatial ability measure demonstrating a lot of unexplained variance (M. V. Likhanov et al., 2018; Rimfeld et al., 2017). It might be that using other measures of spatial ability—tapping into the other components of spatial ability, for example, navigation (Redhead et al., 2023), and the updated Nostalgia Content Questionnaire—will strengthen the current weak association between spatial ability and SNS score.

The current study also showed that more work is needed to further improve nostalgia experience scales. In particular, new instruments should systematically probe events cued by visual, auditory, olfactory, gustatory, etc., triggers—as different senses evoke memories with different qualities (Lopis et al., 2021; Schlintl et al., 2023). For example, odors tend to elicit older, more emotional childhood memories (Lopis et al., 2021). The new instruments should use 5- to 7-point Likert scales so participants can rate phenomenological features (vividness, emotion, trigger intensity, or frequency of experience)—similar to existing measures of autobiographical memory (e.g., Rubin et al., 2003). Such comprehensive measures will allow further investigation into nostalgia profiles; for example, whether the triggers and content of nostalgic experience are in some way matched. It could be that people whose nostalgia is often triggered by a particular modality (e.g., taste) will experience more nostalgia or more vivid nostalgia in this modality. For example, research showed that matching a smell at recall with the memory’s original context yields higher vividness than a mismatched cue (Chu & Downes, 2002). In addition, our analysis showed that some items should be dropped from the new instrument as they showed little variance in responses. For example, >90% of respondents selected colored vs. black–white option in “The surrounding environment in my nostalgic memories is usually…” question. In addition, future research could add items tapping into “proneness to spatial nostalgia” to the Nostalgia Proneness Questionnaire, e.g., asking whether a person returns to significant places from the past often during nostalgic experiences.

To summarize, further research with new methods will shed light on whether nostalgic experience profiles exist, how they are distributed in the population, and whether these differences in profiles are related to spatial ability and well-being.

## Figures and Tables

**Table 1:** Nostalgia Content Questionnaire.

Variable Name	Question	Response Options	Coding in SNS *
Nost 2	The last time I indulged in nostalgic memories	a—Never/a long time ago	
		b—A month ago	
		c—A week ago	
		d—A couple of days ago	
		e—Today	
Nost_3	**The objects in my nostalgic memories usually…**	**a—Cannot be clearly described**	**0**
		**b—Have some form and shape**	**1**
		**c—Have a clear form and shape**	**2**
Nost_4	The surrounding environment in my nostalgic memories is usually…	a—Black and white	
		b—Colored If you selected this option, the question Nost4_2 will be presented	
Nost_4_2	The colors in my nostalgic memories are…	a—Warm colors	
		b—Cold colors	
		c—As they were in reality	
		d—Vary from time to time	
Nost_5	I usually experience nostalgic memories when… (choose all that apply)	a—I smell something from the past	
		b—I hear sounds that remind me of the past	
		**c—I see familiar images**	**1**
		d—I feel special touches from the past	
		e—I taste something that triggers memories	
		f—I feel sad	
		**g—I return to places tied to memories**	**1**
Nost_6	**Most often during nostalgic memories, I… (choose all that apply)**	a—Smell something (e.g., baked goods)	
		b—Hear something (e.g., a voice)	
		**c—Imagine only visual images**	**1**
		d—Feel touch (e.g., a person, breeze)	
		e—Other (please specify)	
Nost_7	**In my nostalgic memories, I return…**	**a—Mostly to the same place**	**1**
		**b—Mostly to different places**	**2**
Nost_8	In my nostalgic memories, I am usually…	a—Alone	
		b—With someone else	
Nost_8_2	For those who selected “with someone else” in Nost_8 The people usually present in my nostalgic memories are… (choose all that apply)	a—Family members	
		b—Friends	
		c—Neighbors	
		d—Stranger	
		e—Pets	
		f—Other (please specify)	
Nost_9	**In my nostalgic memories, I…**	**a—Move around dynamically (e.g., inside a house)**	**1**
		**b—Move objects in space**	**1**
		c—Mostly observe passively without moving	
		**d—The space or objects move around me**	**1**
Nost_10	I most often engage in nostalgic memories…	a—Alone	
		b—In conversation with someone	
Nost_11	**In my nostalgic memories, I usually…**	a—Speak words (out loud or to myself)	
		**b—Visualize something without speaking**	**1**

Note: * the formula for calculating Spatial Nostalgia Score (SNS) was as follows: Nost_3 + Nost5c + Nost7 + Nost_9a + Nost_9b + Nost_9d + Nost_11b; questions and response options included in the SNS are in bold.

**Table 2:** Descriptive statistics.

	BFI: Neuroticism	GAD7	Nostalgia Proneness	WHO5	Spatial Ability
Valid	473	440	486	474	445
Mean	2.811	7.314	29.893	13.751	7.265
Std. Deviation	0.889	4.936	9.338	4.845	3.459
Skewness	0.120	0.980	−0.454	−0.178	0.280
Kurtosis	−0.749	0.417	−0.315	−0.562	−0.911
Minimum	1.000	1.000	1.920	2.000	1.000
Maximum	5.000	21.817	48.000	25.000	15.000

## Data Availability

The data and the code used to obtain results reported in the current study are available from the corresponding author upon reasonable request.

## Supplementary Materials

The following supporting information can be downloaded at: https://www.mdpi.com/article/10.3390/bs15121669/s1. Table S1: Frequency table for all variables of Nostalgia Content Questionnaire; Table S2. Results of ANOVA and descriptive statistics for the 4 variables.
