# Supplementary material for: Unfolding Nostalgia: Spatial Visualization, Nostalgia, and Well-Being
Source: Behav Sci (Basel). 2025 Dec 3;15(12):1669. doi: 10.3390/bs15121669 (PMC12729733; doi:10.3390/bs15121669)
Supplement: Supplementary file 1 [file behavsci-15-01669-s001.zip › behavsci-3945674-supplementary.pdf]

Title: **Unfolding Nostalgia: Spatial Visualization, Nostalgia, and Well-Being**

Maxim Likhanov [1,*], Ksenia Bartseva [1], Elena Soldatova [1] and Yulia Kovas [2]

[1] Department of Psychology, Saint Petersburg State University, 7/9 Universitetskaya Nab., 199034 Saint Petersburg, Russia; k.bartseva@spbu.ru (K.B.); e.l.soldatova@spbu.ru (E.S.)
[2] Cognitive Health and Intelligence Centre, Institute for Cognitive Neuroscience, HSE University, 101000 Moscow, Russia; yvkovas@hse.ru
* Correspondence: maximus.minimus@mail.ru

Table S1. Frequency table for all variables of Nostalgia Content Questionnaire

Variable	Question	Response option	Count	Percent
Colors	The surrounding environment in my nostalgic memories is usually...	Colored	444	91.7
		Black and white	40	8.3
Colors2	The colors in my nostalgic memories are…	True colors	286	64.4
		Warm colors	110	24.8
		Different colors	41	9.2
		Cold colors	7	1.6
During: alone	In my nostalgic memories, I am usually	With someone	402	83.1
		Alone	82	16.9
During: hear	Most often during nostalgic memories, ...	Not selected	298	61.6
		Hear something	186	38.4
During: images		Imagine visual images	418	86.4
		Not selected	66	13.6
During: smell		Not selected	310	64
		Smell something	174	36

During: touch		Not selected	398	82.2
		Feel–touch	86	17.8
Family	The people usually present in my nostalgic memories are...	Family member	348	86.6
		Not selected	54	13.4
Friends		Friends	356	88.6
		Not selected	46	11.4
Strangers		Not selected	375	93.3
		Strangers	27	6.7
Neighbors		Neighbors	356	88.6
		Not selected	46	11.4
Pets		Not selected	252	62.7
		Pets	150	37.3
Object clarity	The objects in my nostalgic memories usually...	Sharp objects	255	52.7
		Have some form	193	39.9
		impossible to describe	36	7.4
Return	In my nostalgic memories, I return...	Diff. place	360	74.4
		Same place	124	25.6
Speak or imagine	In my nostalgic memories, I usually...	Imagine something	367	75.8
		Talk to myself	117	24.2
Trig: sound	I usually experience nostalgic memories when	Hear sounds	253	52.4
		Not selected	230	47.6
Trig: images		See images	352	72.9
		Not selected	131	27.1
Trig: places		Return to place	377	78.1
		Not selected	106	21.9
Trig: sad		Not selected	309	64
		Feel sad	174	36
Trig: smell		Smell something	277	57.3
		Not selected	206	42.7

Trig: taste		Not selected	264	54.7
		Taste something	219	45.3
Trig: touch		Not selected	400	82.8
		Feel–touch	83	17.2
Trig: alone	I most often engage in nostalgic memories...	Alone	255	52.7
		In conversation	229	47.3
Movement	In my nostalgic memories, I...	Do not move myself	225	46.5
		Move myself	199	41.1
		Space/objects moves	43	8.9
		Move something	17	3.5

Note: Question 1 was excluded from analysis as it mostly concerned the frequency of nostalgic experiences and correlated quite highly with the Nostalgia Proneness Questionnaire.

Table S2. Results of ANOVA and descriptive statistics for the 4 variables

	Membership	N	Mean	SD	SE	F(df1,df2)
**Spatial ability**	**1**	187	7.22	3.48	0.254	0.95(2,184)
	**2**	157	7.44	3.41	0.272	
	**3**	68	6.75	3.45	0.419	
**WHO5**	**1**	202	13.53	5.06	0.356	0.61(2,191)
	**2**	167	13.86	4.71	0.365	
	**3**	70	14.27	4.88	0.583	
**Nostalgia proneness**	**1**	206	29.07	9.51	0.663	1.46(2,201)
	**2**	171	30.73	9.29	0.710	
	**3**	74	29.76	9.49	1.103	

Table S2. Results of ANOVA and descriptive statistics for the 4 variables

	Membership	N	Mean	SD	SE	F(df1,df2)
**GAD7**	**1**	183	7.55	4.92	0.364	0.12 (2, 179)
	**2**	160	7.30	5.32	0.420	
	**3**	62	7.32	4.40	0.559	
